# Comparative analysis of the organelle genomes of three *Rhodiola* species provide insights into their structural dynamics and sequence divergences

**DOI:** 10.1186/s12870-023-04159-1

**Published:** 2023-03-22

**Authors:** Xiaolei Yu, Pei Wei, Zhuyifu Chen, Xinzhong Li, Wencai Zhang, Yujiao Yang, Chenlai Liu, Shuqi Zhao, Xiaoyan Li, Xing Liu

**Affiliations:** 1 State Key Laboratory of Hybrid Rice, Laboratory of Plant Systematics and Evolutionary Biology, College of Life Sciences, Wuhan University, Wuhan, 430072 Hubei China; 2 Laboratory of Extreme Environmental Biological Resources and Adaptive Evolution, Research Center for Ecology, School of Sciences, Tibet University, Lhasa, Tibet, 850000 China; 3 Biology Experimental Teaching Center, School of Life Science, Wuhan University, Wuhan, 430072 Hubei China

**Keywords:** *Rhodiola*, Organelle genome, Structure dynamic, Sequence evolution, Positive selection, Comparative analysis

## Abstract

**Background:**

Plant organelle genomes are a valuable resource for evolutionary biology research, yet their genome architectures, evolutionary patterns and environmental adaptations are poorly understood in many lineages. *Rhodiola* species is a type of flora mainly distributed in highland habitats, with high medicinal value. Here, we assembled the organelle genomes of three *Rhodiola* species (*R. wallichiana*, *R. crenulata* and *R. sacra*) collected from the Qinghai-Tibet plateau (QTP), and compared their genome structure, gene content, structural rearrangements, sequence transfer and sequence evolution rates.

**Results:**

The results demonstrated the contrasting evolutionary pattern between plastomes and mitogenomes in three *Rhodiola* species, with the former possessing more conserved genome structure but faster evolutionary rates of sequence, while the latter exhibiting structural diversity but slower rates of sequence evolution. Some lineage-specific features were observed in *Rhodiola* mitogenomes, including chromosome fission, gene loss and structural rearrangement. Repeat element analysis shows that the repeats occurring between the two chromosomes may mediate the formation of multichromosomal structure in the mitogenomes of *Rhodiola*, and this multichromosomal structure may have recently formed. The identification of homologous sequences between plastomes and mitogenomes reveals several unidirectional protein-coding gene transfer events from chloroplasts to mitochondria. Moreover, we found that their organelle genomes contained multiple fragments of nuclear transposable elements (TEs) and exhibited different preferences for TEs insertion type. Genome-wide scans of positive selection identified one gene *matR* from the mitogenome. Since the *matR* is crucial for plant growth and development, as well as for respiration and stress responses, our findings suggest that *matR* may participate in the adaptive response of *Rhodiola* species to environmental stress of QTP.

**Conclusion:**

The study analyzed the organelle genomes of three *Rhodiola* species and demonstrated the contrasting evolutionary pattern between plastomes and mitogenomes. Signals of positive selection were detected in the *matR* gene of *Rhodiola* mitogenomes, suggesting the potential role of this gene in *Rhodiola* adaptation to QTP. Together, the study is expected to enrich the genomic resources and provide valuable insights into the structural dynamics and sequence divergences of *Rhodiola* species.

**Supplementary Information:**

The online version contains supplementary material available at 10.1186/s12870-023-04159-1.

## Introduction

Chloroplasts and mitochondria are endosymbiotic organelles with their own genetic information, the former transforming solar energy into carbohydrates and oxygen in green land plants and algae, while the latter providing energy currency to life processes in all eukaryotic species [1]. Plastomes and mitogenomes have many traits in common, such as mutational patterns [2], replication strategies [3], and modes of inheritance [4]. Nevertheless, there are also some large differences between plastomes and mitogenomes in land plants. Generally, plastomes are believed to be a typically quadripartite structure with a highly conserved circular structure, stable gene content, gene order and sequence composition, while mitogenomes are known for their remarkably variable genome size, structural complexity, and ability to incorporate foreign DNA [1]. For example, most plastomes have a quadripartite structure that includes two copies of inverted repeat region separating large and small single-copy regions, and with a size ranging from 100 to 200 kb [5]. But mitogenomes are structurally diverse, not only exist as a single-master circle, but also appear frequently as sub-genomic molecules [6, 7]. The plant mitogenome is also remarkable in genome size, and the difference in genome size between different taxa can even span over a 100-fold range (about 66-11,000 kb) [8, 9]. Nevertheless, much of this knowledge comes from comparisons of remote lineages, probably because mitogenomes of land plants are so scarce that data on closely related species are difficult to obtain.

In most seed plants, both chloroplasts and mitochondria are inherited maternally, with multiple copies in each cell [10, 11]. Maternal inheritance makes only one allele per cell in the genetic information of organelle DNA, while multiple copies allow the organelle DNA to be easily retrieved even when the DNA is low-quantity or degraded. These properties make the organelle genomes widely used in evolutionary biology studies [12, 13], such as inferring phylogenetic relationships [14, 15] or identifying genes associated with environmental adaptation [16, 17]. There are several lines of evidence that sequence variation in plant organelle genomes is frequently adaptive, including cytoplasmic trapping, cytoplasmic effects in local adaptation, and positive selection in chloroplast genes [18–21]. The adaptive contribution of genetic variation in plant organelles can be investigated by looking for the footprints left by positive selection in DNA variation patterns, and comparative genomic analysis provides an opportunity to gain insights into the adaptive evolution of these genomes [22]. Current work on the adaptive contribution of plant organelle genomes has disproportionately focused on the plastomes [23–25]. Therefore, it is necessary to extend these investigations to the mitogenomes.

The *Rhodiola* genus, belonging to the Crassulaceae family, contains about 90 species mainly distributed in high altitudes and cold regions of the Northern Hemisphere [26]. *Rhodiola* species are perennial herbs with well-developed leafy or scaly rhizomes, which have been traditional medicines in Eastern Europe and Asia in the treatment of headaches, hernias, lung disorders, bleeding, burn and soft tissue injuries [27, 28]. Moreover, they are often used as tonics and stimulants to increase physical endurance, work productivity and longevity, as well as energy levels [29]. Preparations of this drug are now part of the official medicine of some countries [28]. In China, the dry roots and rhizomes of *Rhodiola crenulata* (*R. crenulata*) are the only source of Chinese medicine Hongjingtian. The *Rhodiola* species usually grow on gravel-covered slopes or cracks in exposed rock at altitudes of about 3500–5000 m, making it very difficult to collect and study them [30, 31]. To date, many pharmacological effects have been reported for *Rhodiola* species, but few studies have focused on its highland adaptation. Although a recent study has found some footprints of high-altitude adaptation on the *Rhodiola* plastomes [24], it is unclear whether these are also present on mitogenomes, and what role they play in adaptation.

In this study, we assembled and annotated the organelle genomes of three *Rhodiola* species, *R. wallichiana R. crenulata* and *R. sacra*, which were collected from 3,540 to 4,898 m above sea level from the QTP. We then compared the genetic features of their organelle genomes, including genome structure, gene content, repeat sequences, homologous sequences, transposable elements (TEs) and nucleotide substitution rate in protein-coding genes (PCGs). We explored the adaptive contribution of mitogenomes of *Rhodiola* species by detecting the footprints left by positive selection in their DNA sequences. To the best of our knowledge, we assembled here the first mitogenomes in the Crassulaceae family, which could be regarded as reference genomes for subsequent analyses of the Crassulaceae family. Specially, our study attempts to (1) gain insights into the evolutionary patterns of chloroplasts and mitochondria among three *Rhodiola* species, and (2) evaluate the potential role of the mitogenomes during long-term adaptation of *Rhodiola* species to highland environments.

## Results

### Genome organization of three ***Rhodiola ***organelles

**Fig. 1:**
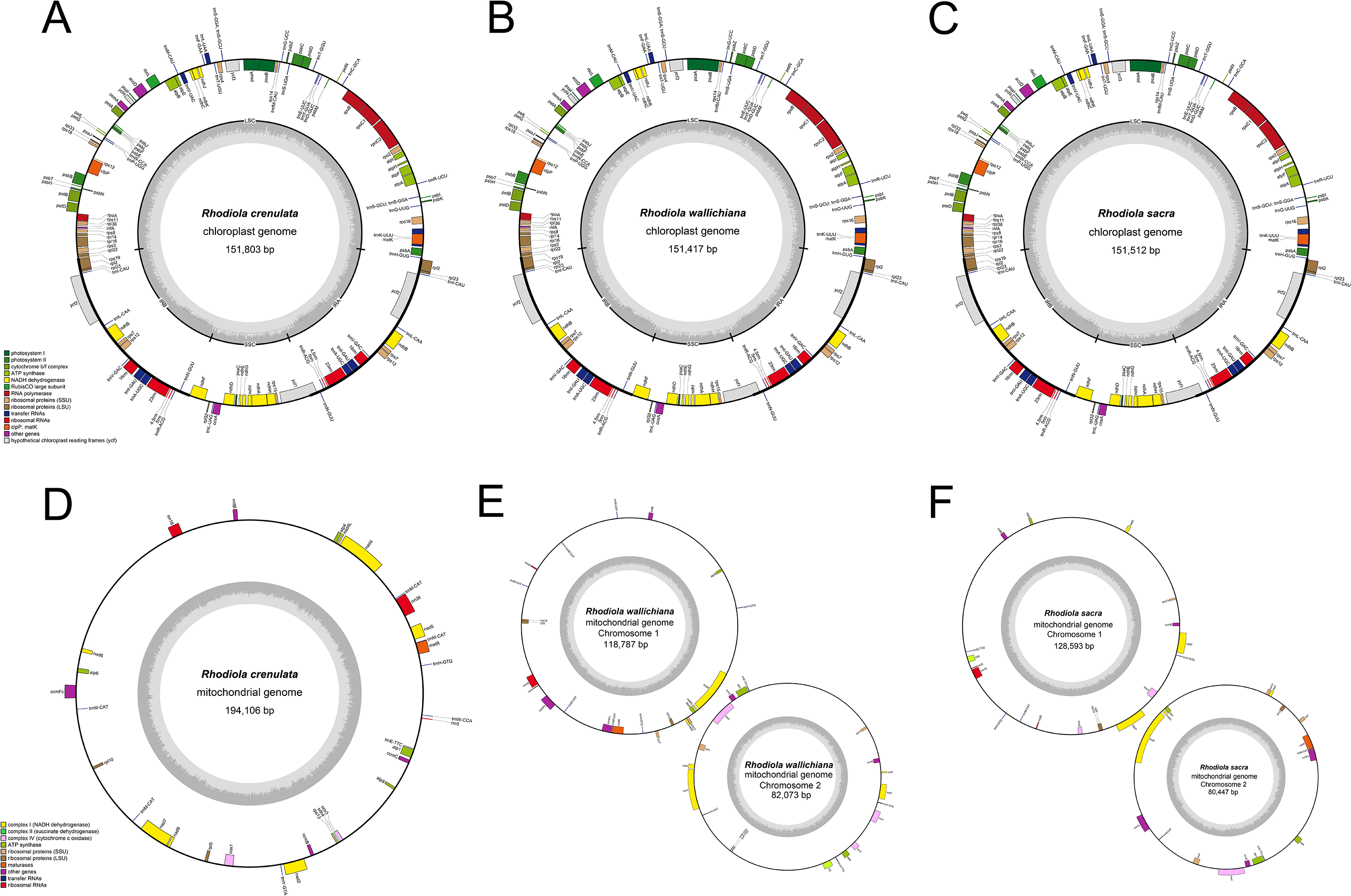
Genome map of the organelle genomes of three *Rhodiola* species. (**A**-**C**) Plastome. (**D**-**F**) mitogenome. Among them, *R. crenulata* mitogenome have a single-ring structure, while R. *wallichiana* and *R. sacra* mitogenomes have a double-ring structure

 The complete plastomes of *R. crenulata*, *R. wallichiana* and *R. sacra* were 151,803, 151,417 and 151,512 bp in length, respectively (Fig. 1; Table 1). All of these genomes exhibited a typical quadripartite structure, including a pair of inverted repeats (IRs) (25,851 bp in *R. crenulata*, 25,847 bp in *R. wallichiana* and 25,882 bp in *R. sacra*) separated by small single-copy region (17,054 bp in *R. crenulata*, 17,052 bp in *R. wallichiana* and 17,037 bp in *R. sacra*) and large single-copy region (83,047 bp in *R. crenulata*, 82,671 bp in *R. wallichiana* and 82,711 bp in *R. sacra*). The *Rhodiola* plastomes showed consistent gene content and order, containing 129 genes consisting of 85 protein-coding genes, 36 tRNA genes and 8 rRNA genes (Table S1).

We found an interesting phenomenon that the *R. crenulata* mitogenome was assembled into one circular chromosome (Fig. 1), whereas the *R. wallichiana* and *R. sacra* mitogenomes were assembled into two circular chromosomes, with an atypical multi-ring conformation. Then, Illumina reads mapping to the mitogenome sequences with Bowtie 2 was applied as an additional genome structure verification step (Figure S1). The results showed that the mitogenome assembly of three *Rhodiola* species was complete with a relatively homogenous coverage. The circular chromosome of *R. crenulata* mitogenome was 194,106 bp in length, containing 22 PCGs, 8 tRNA and 3 rRNA genes (Table S2). The total length of *R. wallichiana* mitogenome was 200,860 bp, with the larger chromosome of 118,787 bp and the smaller one of 82,073 bp. Similar to *R. wallichiana*, the *R. sacra* mitogenome was 209,040 bp in length, with the larger one of 128,593 bp and the smaller one of 80,447 bp. Both the *R. wallichiana* and *R. sacra* mitogenomes contained 27 PCGs and 3 rRNA genes scattered across their two chromosomes (Table S3 and Table S4), except that the number of tRNA genes was different between them (13 in *R. wallichiana* and 7 in *R. sacra*) (Table 1).

**Table 1:** Genomic characteristics of three *Rhodiola* species

Species	Genome	Genome size (bp)	Number of genes	Protein genes	tRNA genes	rRNA genes
*R. crenulata*	Plastome	151,803	129	85	36	8
*R. wallichiana*	Plastome	151,417	129	85	36	8
*R. sacra*	Plastome	151,512	129	85	36	8
*R. crenulata*	Mitogenome	194,106	33	22	8	3
*R. wallichiana*	Mitogenome chr1	118,787	24	14	7	3
	Mitogenome chr2	82,073	19	13	6	0
*R. sacra*	Mitogenome chr1	128,593	19	12	4	3
	Mitogenome chr2	80,447	18	15	3	0

### Phylogeny and lineage-specific chromosome fission and gene lose

**Fig. 2:**
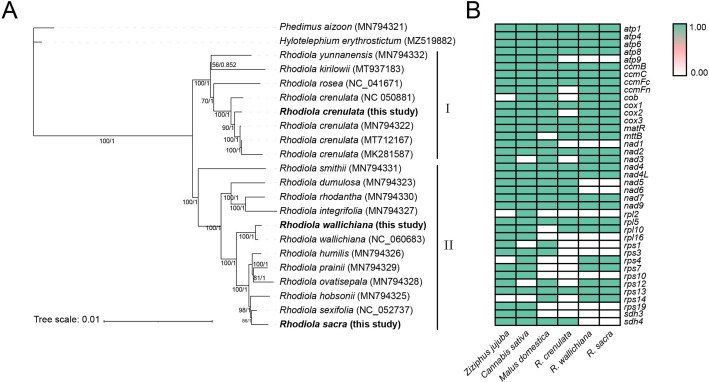
Plastome-based phylogeny and mitogenome-based gene gain and loss. (**A**) Phylogenetic tree of *Rhodiola* species based on 79 shared PCGs in plastomes. Numbers below the lines represent ML bootstrap proportions/ BI posterior probabilities. (**B**) Comparison of mitochondrial PCGs between *Rhodiola* species and other species of the order Rosales

To explore the phylogenetic position of three *Rhodiola* species (*R. crenulata*, *R. wallichiana* and *R. sacra*) within the *Rhodiola* genus, we conducted the phylogenetic analysis based on 79 shared PCGs of 22 plastomes. ML and BI trees had a consistent typology, and most branches had high node support values (Fig. 2A). As expected, our newly assembled plastomes of *R. crenulata* and *R. wallichiana* clustered together with their previously reported plastomes. On the whole, all *Rhodiola* species were divided into two clades, with *R. crenulata* in cladeI, *R. wallichiana* and *R. sacra* in cladeII. The phylogenetic relationship among our three *Rhodiola* species was described as (*R. crenulata* + (*R. wallichiana* + *R. sacra*)), suggesting that *R. wallichiana and R. sacra* were more closely related than *R. crenulata.* This result was in agreement with previous study based on five barcoding markers (*rbcL*, *matK*, *trnH-psbA*, *trnL-F* and internal transcribed spacer) [31].

With phylogenetic results, we inferred that the multi-chromosome structure of *Rhodiola* mitogenomes might be lineage-specific, as *R. crenulata* mitogenome in cladeI had a single-ring structure, while *R. wallichiana* and *R. sacra* mitogenomes in cladeIIhad a double-ring structure. Additionally, we found substantial loss events of PCGs in *Rhodiola* mitogenomes (Fig. 2B). By comparison of mitochondrial PCGs between *Rhodiola* species (22–27 PCGs) and other species of the order *Rosales* (approximately 35 PCGs), we found that many PCGs had been completely lost from *Rhodiola* species’ mitogenomes, including *atp9*, *nad1*, *rpl2*, *rpl16*, *rps1*, *rps3*, *rps19* and *sdh3*. Moreover, it seemed that gene loss events were also lineage-specific, with *R. wallichiana* and *R. sacra* in one clade retaining the same number of PCGs, while some of them, such as *ccmFn*, *cob* and *cox2*, were absent in *R. crenulata* in the other clade.

### Analyses of genomic syntenic regions and rearrangements

**Fig. 3:**
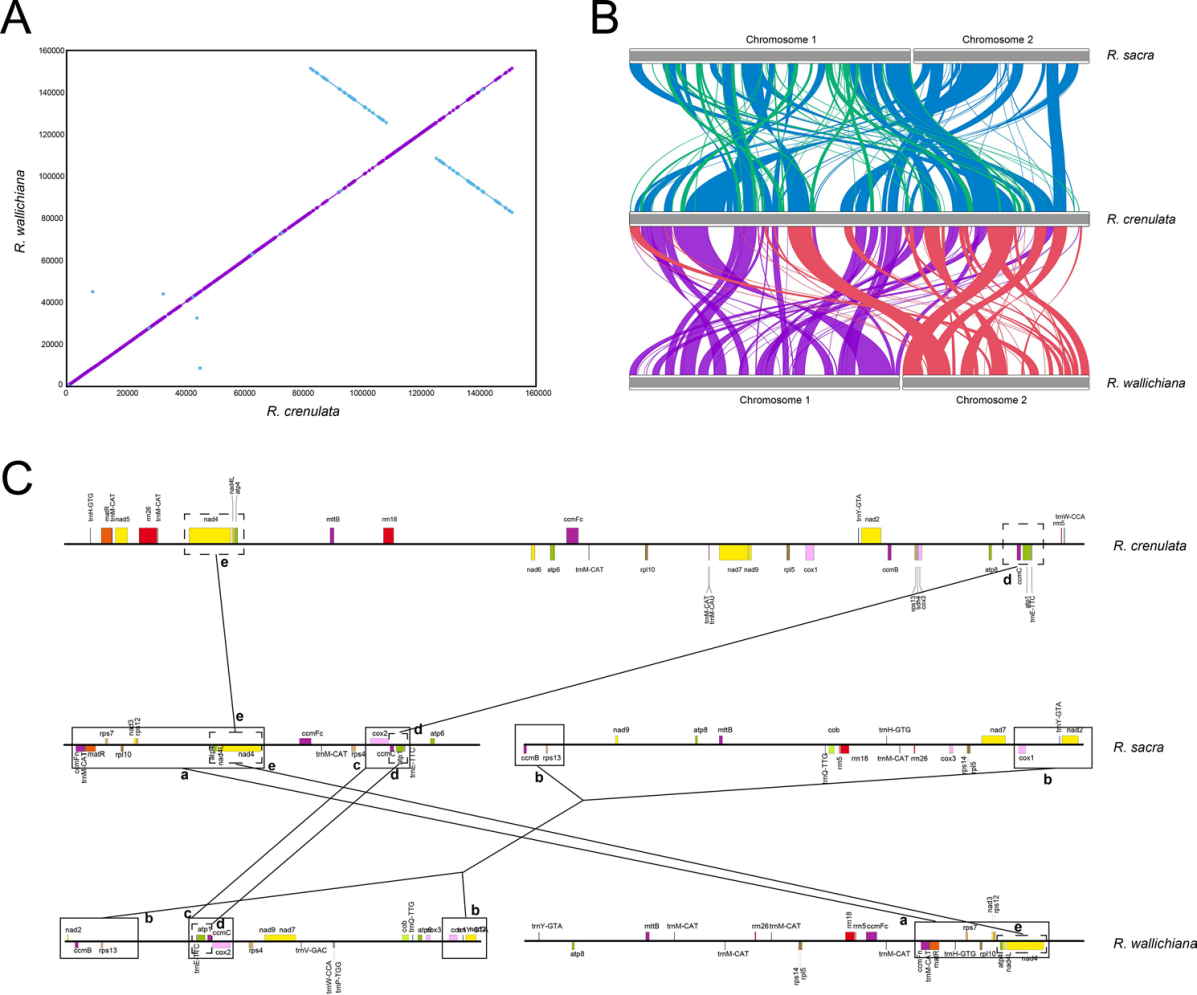
Syntenic analyses of organelle genomes among three *Rhodiola* species. (**A**) Syntenic regions between *R. crenulata* and *R. wallichiana* plastomes. (**B**) Syntenic regions of mitogenomes among three *Rhodiola* species. (**C**) Conserved gene blocks among the mitogenomes of three *Rhodiola* species. Block (**a**) contains genes *ccmFn*-*trnM*-*matR*-*trnH*-*rps7*-*rpl10*-*nad3*-*rps12*-*atp4*-*nad4L*-*nad4*. Block (**b**) contains genes *cox1*-*trnY*-*nad2*. Block (**c**) contains genes *trnE*-*atp1*-*ccmC*-*cox2*. Block (**d**) contains genes *trnE*-*atp1*-*ccmC*. Block (**e**) contains genes *atp4*-*nad4L*-*nad4*

Synteny block analysis is often used to determine genome evolution among related species. To explore the genome structure evolution of *Rhodiola* species, we analyzed the syntenic regions among their organelle genomes. The plastome structures of *R. crenulata*, *R. wallichiana* and *R. sacra* showed a high degree of collinearity, with almost the entire genome being a collinear region except for the two 25-kb inversions which resulted from inverted repeat regions, suggesting evolutionary conservation of these plastomes at the genome-scale level (Fig. 3A and Figure S1). Nevertheless, the mitogenomes of three *Rhodiola* species exhibited even greater complexity than their plastomes (Fig. 3B). We totally identified 74 collinear blocks (134,109 bp) between the mitogenomes of *R. crenulata* and *R. wallichiana* (Table S5), 90 collinear blocks (137,068 bp) between the mitogenomes of *R. crenulata* and *R. sacra* (Table S6), as well as 94 collinear blocks (146,834 bp) between the mitogenomes of *R. wallichiana* and *R. sacra* (Table S7). These collinear blocks were dispersed throughout their mitogenomes, suggesting multiple structural rearrangement events across *Rhodiola* mitogenomes. We then compared the genomic locations of all orthologous genes in the three mitogenomes to explore whether rearrangement events would disrupt the gene clusters (Fig. 3C). We found that although many rearrangements disrupted the gene clusters across three *Rhodiola* mitogenomes, some gene clusters were also retained and exhibited lineage-specific features. For example, three larger gene clusters (block a, b and c) were presented between *R. wallichiana* and *R. sacra*, of which only two smaller gene clusters (block d and e) were preserved in all three species.

### Analyses of repeat elements

**Fig. 4:**
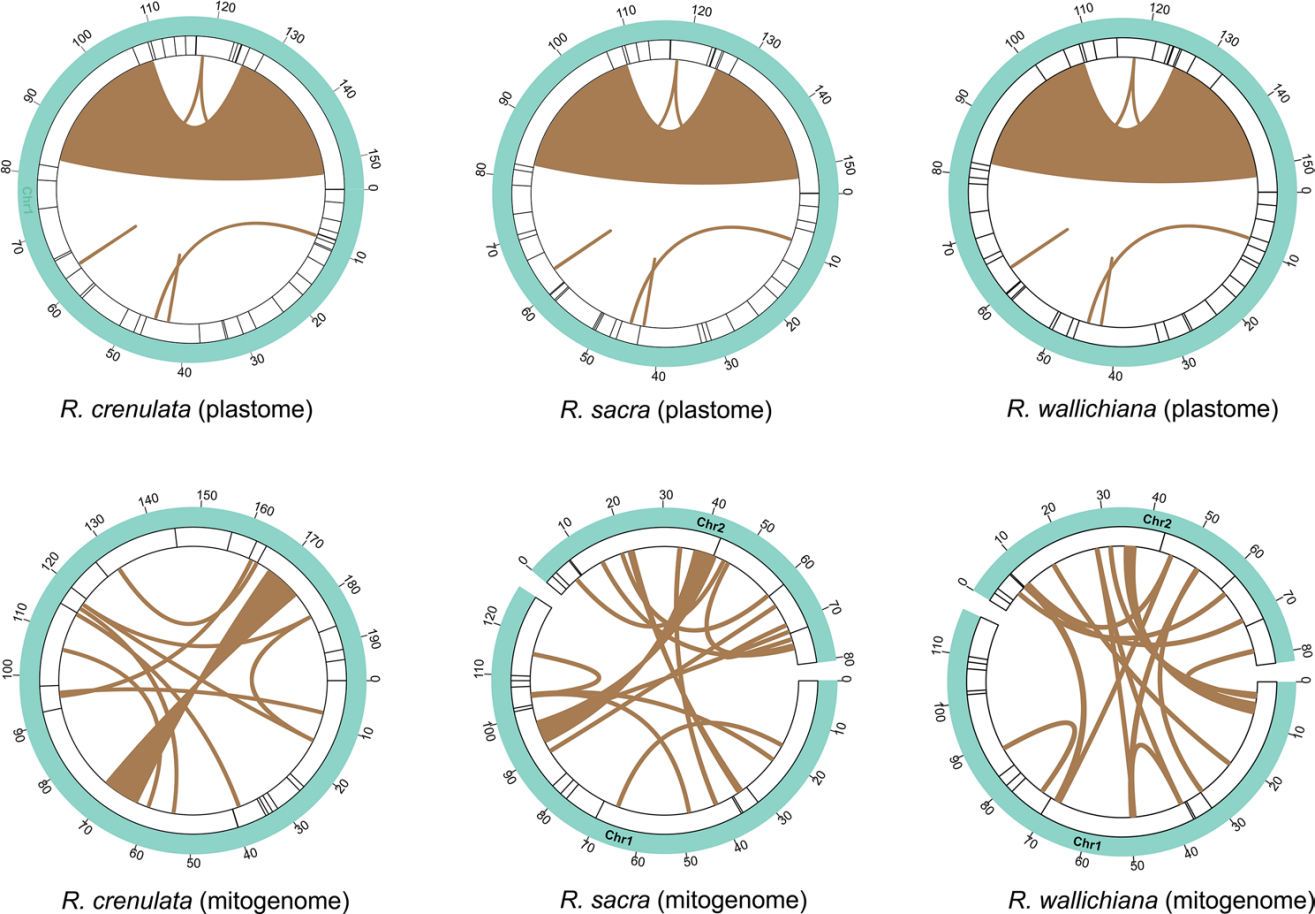
Repeat sequences in the organelle genomes of three *Rhodiola* species. The links on the innermost circles represent repeats identified by BLASTn. Bars on the second circles represent SSRs.

Repetitive sequences play an important role in genomic rearrangement, recombination, and sequence divergence in plant organelle genomes [32–34]. We identified repetitive sequences in the *Rhodiola* organelle genomes by blasting the genome against itself and deleting results that matched itself. For the plastome, we did not identify any large repeats greater than 1 kb, except for two IR regions (Fig. 4 and Table S8). The repeat sizes of these small repeats are all below 100 bp (40–94 bp in *R. crenulata*, 40–96 bp in *R. sacra* and 40–94 bp in *R. wallichiana*). BLASTn analysis of the *Rhodiola* mitogenomes against themselves respectively revealed 8,656 bp, 6,702 bp, and 3,469 bp of repetitive DNA in *R. crenulata*, *R. sacra*, and *R. wallichiana*, ranging from 29 to 8,145 bp, 29 to 5,186 bp, 29 to 3,469 bp (Fig. 4 and Table S8). Plant mitochondrial genomes are known to be rich in repetitive sequences that can participate in frequent homologous recombination. These repetitive sequences are thought to be responsible for the structural diversification of the plant mitogenome [35]. In this study, we found that most repetitive sequences occur between the two chromosomes and possess high sequence similarity. For example, 92.7% of repetitive sequences occurred between the two chromosomes of *R. sacra* (sequence similarity ≥ 99.846%), and this proportion was 93.4% in *R. wallichiana* (except for a very low sequence similarity of 74.222%, the sequence similarity of the rest of the repeats is above 90%) (Table S8). This suggests that these repeats may mediate the formation of multichromosomal structure in the mitogenomes of *Rhodiola*, and that such multichromosomal structure may have recently formed.

We also identified simple sequence repeats (SSRs), also known as microsatellites, which are 1- to 6-bp repetitive sequences that are widely distributed throughout a genome [36]. Their high polymorphism and codominant inheritance make them popular molecular markers in population genetic studies [37, 38]. The results showed that SSRs were much more abundant in the plastome than in the mitogenome. For example, a total of 438 bp (42 fragments) SSRs were identified in the *R. crenulata* plastome, whereas only 211 bp (20 fragments) SSRs were identified in the *R. crenulata* mitogenome (Table S9). Likewise, we identified 434 bp (41 fragments) and 445 bp (44 fragments) SSRs in the plastomes of *R. sacra* and *R. wallichiana*, respectively, while only 194 bp (20 fragments) SSRs were identified in their mitogenomes (Table S9). Overall, our study indicates that the SSRs in the *Rhodiola* plastomes are more abundant than those in the mitogenomes, making them more suitable for population genetic studies of *Rhodiola* species.

### Analyses of homologous sequences between plastomes and mitogenomes

**Fig. 5:**
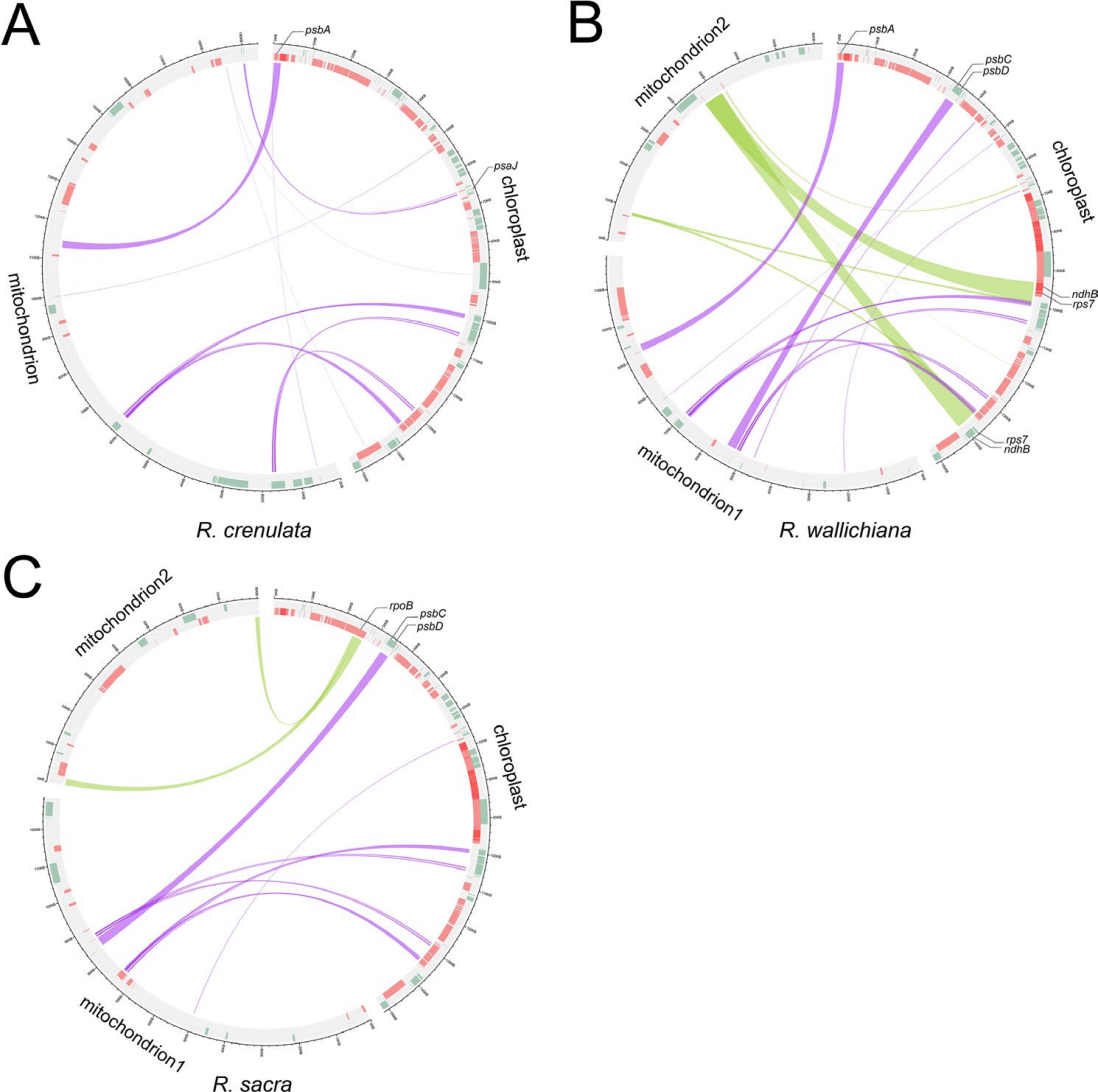
Homology sequences between plastome and mitogenome. On the circle plot, the red bars represent genes in the counter clockwise direction and the green bars represent genes in the clockwise direction. The shaded links represent identified homologous sequences. In homologous sequences, intact PCGs are highlighted with a broken line. (**A**) *R. crenulata*. (**B**) *R. wallichiana*. (**C**) *R. sacra*

We searched for homologous sequences between plastomes and mitogenomes in three *Rhodiola* species to identify potential gene transfer events. We identified 18–26 fragments of homologous sequences between plastomes and mitogenomes from three *Rhodiola* species, with the total length ranging from 4,752 to 22,452 bp. Notably, several intact plastome-derived PCGs were identified in their mitogenomes, including *psbA*, *psbJ*, *psbC*, *psbD*, *ndhB*, *rps7*, *rpoB* (Fig. 5). Of these PCGs, *psbA* was transferred in both *R. crenulata* and *R. wallichiana*, while *psbC* and *psbJ* were transferred in both *R. wallichiana* and *R. sacra.* These results demonstrated extensive intracellular DNA transfer from chloroplast to mitochondria in *Rhodiola* species. Nevertheless, we did not find intact mitogenome-derived PCGs identified in their plastomes.

### Identification of TEs in ***Rhodiola*** organelle genomes

The *Rhodiola* plastomes contained many TEs fragments with a total length ranging from 13,906 (*R. wallichiana*) to 14,392 bp (*R. crenulata*), accounting for 9.2-9.5% of the total length of the plastomes (Table 2). These TEs fragments could be classified into two main classes, DNA transposons and retrotransposons. Among them, DNA transposons accounted for a large part, with 9,136 bp (64.5%) in *R. crenulata*, 8,811 bp (63.4%) in *R. wallichiana*, 8,623 bp (61.2%) in *R. sacra*. Compared with plastomes, the identified TEs were relatively infrequent in mitogenomes. We only identified a total length of 6,630 to 11,683 bp TEs in three *Rhodiola* mitogenomes, accounting for 3.3-6% of the total length of the mitogenomes (Table 2). In contrast to the plastomes, retrotransposons account for a large proportion of TEs in the mitogenomes, with 8,302 bp (71.1%) in *R. crenulata*, 3,676 bp (55.4%) in *R. wallichiana*, 5,261 bp (59.8%) in *R. sacra*. Additionally, there were also some differences between plastomes and mitogenomes in terms of the types of TEs. For example, the retrotransposons SINE and SINE2/tRNA were only identified in three plastomes, while the retrotransposons RTE and DNA transposons Mariner/Tc1 were only detected in three mitogenomes.

**Table 2:** Comparation of TEs in three *Rhodiola* organelle genomes

Type	*R. crenulata*		*R. wallichiana*		*R. sacra*	
	Plastome	Mitogenome	Plastome	Mitogenome	Plastome	Mitogenome
DNA transposon (bp)	9,136	3,381	8,811	2,954	8,623	3,537
EnSpm/CACTA	1,160	414	1,062	237	963	716
Harbinger	130	75	80	64	/	73
Helitron	3,442	446	3,192	404	3,094	631
Mariner/Tc1	/	140	/	174	/	79
MuDR	3,385	2,081	3,503	1,843	3,600	1,420
hAT	1019	225	889	232	907	618
LTR Retrotransposon (bp)	5,027	7,333	4,859	3,302	5,145	4,887
Copia	3,523	3,898	3,477	1,760	3,524	1,975
Gypsy	1,321	3,435	1,199	1,501	1,375	2,912
Non-LTR Retrotransposon (bp)	229	969	236	374	320	374
L1	174	845	181	326	265	326
Penelope	/	76	/	/	/	/
Naiad/Chlamys	/	76	/	/	/	/
RTE	/	48	/	48	/	48
SINE	55	/	55	/	55	/
SINE2/tRNA	55	/	55	/	55	/
Total	14,392	11,683	13,906	6,630	14,088	8,798
Ratio	9.50%	6%	9.20%	3.30%	9.30%	4.20%

Note: The ratio was obtained by dividing the transposon sequence length by the genome length

### Comparison of nucleotide substitution rates in ***Rhodiola*** organelle genomes

**Fig. 6:**
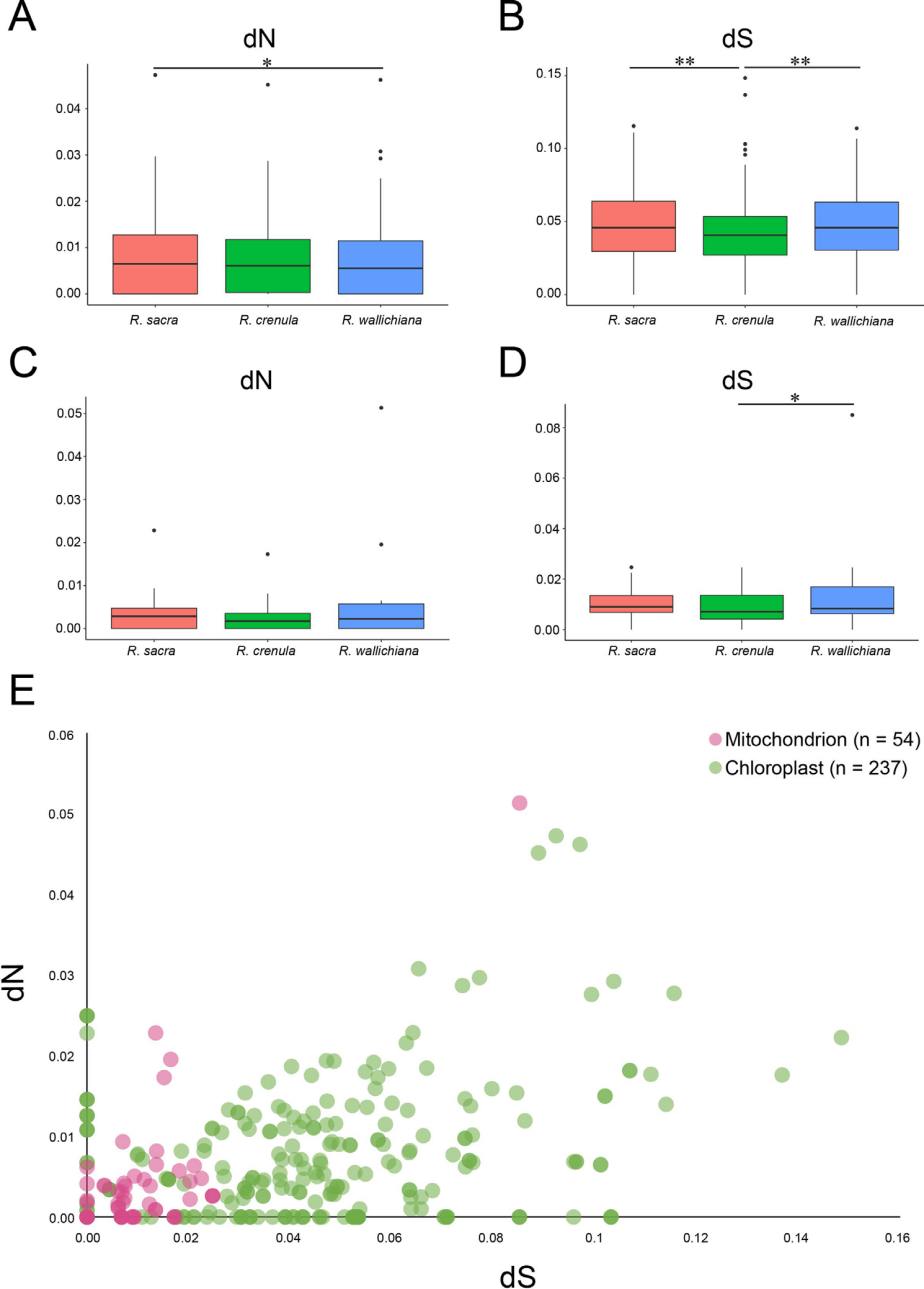
Variation in sequence divergence across species and organelles. (**A**) Comparison of dN values across three *Rhodiola* plastomes. (**B**) Comparison of dS values across three *Rhodiola* plastomes. (**C**) Comparison of dN values across three *Rhodiola* mitogenomes. (**D**) Comparison of dS values across three *Rhodiola* mitogenomes. (**E**) Comparison of dN and dS values across organelles

The synonymous and nonsynonymous divergences are used to compare the rates of sequence evolution across species and organelles. Synonymous divergence generally reflected the mutation input, while nonsynonymous divergence always mirrored the degree of selective pressure [39, 40]. We hypothesized that some organelle genes of *Rhodiola* species distributed at high altitudes might have undergone adaptive evolution to adapt to alpine environments compared with species at low altitudes. Between three *Rhodiola species* and an outgroup *Hylotelephium erythrostictum* (generally distributed in relatively low altitudes of 400-1,800 m [41]), we calculated the dN, dS of 79 shared PCGs from plastomes (Table S10) and 17 shared PCGs from mitogenomes (Table S11). We first compared synonymous and nonsynonymous divergence across species. We found that the dN of chloroplast genes in *R. sacra* was significantly higher than that in *R. wallichiana* (P < 0.05), suggesting that the evolution rate of the chloroplast gene was faster in *R. sacra* than in *R. wallichiana* (Fig. 6A). Although the dN of chloroplast genes in *R. crenulata* was higher than that in *R. wallichiana*, the difference was not significant (P > 0.05). Furthermore, we compared the dS of chloroplast genes across three *Rhodiola* species and found that both *R. sacra* and *R. wallichiana* exhibited significant higher dS value than *R. crenulata* (Fig. 6B). This may due to that both *R. sacra* and *R. wallichiana* are in the same phylogenetic clade (cladeII), resulting in their similar nucleotide mutation rates and significantly different nucleotide mutation rates from *R. crenulata*, which is in cladeI. We then compared synonymous and nonsynonymous divergence of mitochondrial genes across three *Rhodiola* species (Fig. 6C and D). We found that the dN and dS values of three *Rhodiola* species were hardly significantly different, except that the dS of *R. wallichiana* was significantly higher than that of *R. crenulata*.

A comparison of dN and dS values of all PCGs across organelles showed that the sequence divergence of the mitochondrial genes appears to be lower than that of the chloroplast genes and especially the synonymous divergence, although there are also some overlaps in rates between genes across the two organelles (Fig. 6E). We further compared the differences of dN and dS of PCGs between chloroplast and mitochondria using the Mann-Whitney U test and found that both dS and dN values of chloroplast PCGs were significantly greater than those of mitochondrial PCGs (p-value < 2.2e-16 for dS and p-value = 0.0002776 for dN). This result suggested that mitochondrial genes generally evolve at slower rates than chloroplast genes, despite similar evolution rates for some genes between the two organelles. The branch-site model analysis detected a positively selected gene, *matR*, on mitogenome (Table 3). Positive selection for this gene suggested that it might be driven by natural selection in high-altitude environments.

**Table 3:** Positively selected genes and sites detected in the mitogenomes of *Rhodiola* species

Genome	gene	Positive sites	LRT p-value
Mitogenome	*matR*	147, D, 0.957; 201, N, 0.994; 534, S, 0.567; 655, V, 0.842	0.0000001

Note: In positive sites, integers represent the position of the site, letters represent the type of amino acid, and decimals represent the posterior probability

## Discussion

### The contrasting evolutionary pattern between plastomes and mitogenomes in three ***Rhodiola*** species

The plastomes and mitogenomes share many properties, such as circular chromosome structure (in most cases), uniparental inheritance, and frequent gene transfers from organelle to the nuclear genome [23, 42]. The parallel evolution of these common features within the same cell suggests that the two organelle genomes may have similar evolutionary trajectories. However, like in other angiosperms, our results revealed that three *Rhodiola* species showed obviously contrasting evolutionary pattern between plastomes and mitogenomes, with the former possessing more conserved genome structure but faster evolutionary rates of nucleotide, while the latter exhibiting structural diversity but slower rates of nucleotide substitution (Figs. 3 and 6). Such contrasting genetic features between plastomes and mitogenomes may be explained by differences in repair mechanisms that can shape the organelle’s genome architecture [23]. In fact, the contrasting evolutionary pattern between plastomes and mitogenomes have been reported from various land plants, but with the exception of Selaginellaceae, where the two organelle genomes exhibit parallel genetic features in nucleotides composition, elevated substitution rates and complicated organization [43]. These results suggest that the evolutionary pattern of these two organelles is lineage specific, thus our study provides valuable insights into the evolutionary patterns of plastomes and mitogenomes in *Rhodiola* species.

### Lineage-specific chromosome fission, gene loss and structural rearrangement in ***Rhodiola*** mitogenomes

In this study, we also found some lineage-specific features in *Rhodiola* mitogenomes, including chromosome structure, gene content and structural rearrangements (Figs. 1, 2 and 3). We found lineage-specific chromosome fission in *Rhodiola* species, where *R. crenulata* mitogenome in cladeI had a single-ring structure, while *R. wallichiana* and *R. sacra* mitogenomes in cladeII had a double-ring structure (Fig. 2A). Although the multi-chromosomal mitogenomes have been reported in many species, including ferns, gymnosperms, eudicots, and monocots [44], it is rare to find lineage-specific chromosome fission between very closely related species. Thus, this result is expected to enrich our understanding of the multi-chromosomal evolutionary pattern of plant mitogenomes.

Chromosome fission can often lead to lineage-specific gene loss events at the genome-wide level [45]. However, in this study, although the gene loss events were lineage-specific, more genes were lost in *R. crenulata* without chromosomal fission (Fig. 2B), suggesting that the loss of mitochondrial genes in *Rhodiola* did not appear to be caused by chromosomal fission, other causes, such as gene transfer events [46], might also lead to the loss of mitochondrial genes in mitogenome. Nonetheless, our study at least showed that some genes (*ccmFn*, *cob* and *cox2*) that were absent in cladeI (*R. crenulata*) but retained in cladeII (*R. wallichiana* and *R. sacra*) were lost in *R. crenulata* after the divergence of cladeI and cladeII. In fact, mitochondrial gene loss, especially the ribosomal protein genes and succinate dehydrogenase genes *sdh3* and *sdh4*, is a relatively frequent and ongoing phenomenon in angiosperms, and most of these mitochondrial gene losses are probably the consequence of gene transfer to the nucleus [47]. In our study, we found that five ribosomal protein genes (*rpl2*, *rpl16*, *rps1*, *rps3*, *rps19*) and a succinate dehydrogenase gene *sdh4* were lost in *Rhodiola* species, suggesting that these genes may have been functionally transferred to nucleus in *Rhodiola* species.

In addition to gene loss, frequent structural rearrangement is another characteristic that plant mitogenomes possess. It has been reported that angiosperm mitogenomes differentiate rapidly at the structural level and show loss of synteny even when compared with closely related species [48–50]. Our study also demonstrated multiple events of structural rearrangement across *Rhodiola* mitogenomes (Fig. 3B), which broke up most gene clusters on the mitogenomes of *Rhodiola* species. Nevertheless, some conserved gene clusters were also retained and exhibited lineage-specific features (Fig. 3C). Since that *R. wallichiana* and *R. sacra* belong to cladeI and *R. crenulata* to cladeII, these results suggest that the degree of structural rearrangement is associated with species differentiation (i.e., genomic synteny decays with time since divergence).

### Evidence for intracellular DNA transfer in the ***Rhodiola*** organelle genomes

A striking feature of plant mitogenome evolution is the frequent insertion of foreign DNA via intracellular transfer of chloroplast and nuclear DNA or horizontal transfer of mitochondrial DNA. For example, about 13% (∼130 kb) of the *zucchini* mitochondrial DNA is represented by chloroplast- and nuclear-derived sequences, although almost all of which are noncoding [51]. Other angiosperms, like *Plantago* and *Ternstroemia* species, contain mitochondrial gene mosaics, formed by horizontal transfer and gene conversion between native and foreign homologs [52, 53]. In this study, we found intracellular DNA transfer events from chloroplast to mitochondria that were widespread in *Rhodiola* species. Notably, several intact chloroplast-derived PCGs were identified in their mitogenomes, whereas no intact mitochondrial-derived PCGs were identified in their plastomes (Fig. 5). This indicates that the intracellular DNA transfer from chloroplast to mitochondria is a unidirectional process in *Rhodiola* species. In fact, the transfer of chloroplast DNA to mitochondrial DNA is reported in most studies, but with few exceptions [54, 55], the reverse transfer event, from mitochondrial DNA to chloroplast DNA, is rarely reported. Thus, it is generally believed that chloroplasts are impenetrable to foreign DNA [56, 57]. However, new data from various angiosperms revealing nucleus-to-chloroplast DNA migration events indicate that nuclear DNA appears to be more readily translocated into chloroplast DNA than mitochondrial DNA [58, 59].

Nuclear TEs are a class of DNA sequences widely present in plant nuclear genomes and play important roles in genome evolution [60]. They can be divided into two main classes based on their transposition mechanism, retrotransposons and DNA transposons [61]. In this study, we identified multiple fragments of nuclear TEs in *Rhodiola* organelle genomes, and the proportion of nuclear TEs identified in the plastome was generally higher than that in the mitogenome (Table 2). This shows that chloroplast DNA seems to accept nuclear DNA more easily than mitochondrial DNA. Moreover, we found that chloroplasts and mitochondria appear to exhibit different preferences for nuclear TEs insertion type, i.e., chloroplasts prefer DNA transposons, while mitochondria prefer retrotransposons. In plant nuclear genomes, retrotransposons are generally easier to replicate and spread than DNA transposons due to their different transposition mechanism, resulting in more abundant retrotransposons than DNA transposons in nuclear genomes [62]. However, we observed a distinct pattern in plastomes, where their DNA transposons were generally more abundant than retrotransposons. We encourage follow-up studies to focus on whether this phenomenon is prevalent in land plants and the mechanisms behind it.

### Signal of positive selection in ***Rhodiola*** mitogenomes

The Qinghai–Tibetan Plateau (QTP) is the highest plateau in the world, characterized by extreme environments such as hypoxia, low temperature and strong solar radiation [63]. Over a long evolutionary history, resident plants have developed specific mechanisms at the molecular level to adapt to the extreme environments of QTP [63]. Signals of positive selection are often considered to be the adaptive footprints of a species in which new mutations favor the species’ survival [64]. Thus, we seek to find the footprints of *Rhodiola* species for high-altitude adaptation by examining positive selection signals of PCGs in *Rhodiola* species. A recent study has documented the adaptive footprints of *Rhodiola* species, in which three positively selected genes (*ndhA*, *ndhH* and *rpl16*) were identified in the plastomes [24]. However, it is not clear whether such positive selection signals also present in the *Rhodiola* mitogenomes. Our study detected a mitochondrial gene *matR* that was positively selected (Table 3). *matR* is closely related to maturases encoded by group II introns in bacteria and has been retained as a conserved ORF in mitogenomes of nearly all angiosperms [47]. Although its biological role remains largely unknown, a recent study has shown that it is involved in the splicing of many group II-containing pre-RNAs in mitochondria and may play a critical role during the early stages of plant development [65]. This splicing activity is critical for the efficient expression of mitochondrial genes and the proper functioning of the mitochondrial electron transport chain, which in turn is important for stress response in plants. For example, the activity of *matR* protein is essential for the maturation of *NAD1* and the synthesis of complex I biogenesis, which are crucial for plant growth and development, as well as for respiration and stress responses [66]. So, we hypothesize that the *matR* gene may contribute to *Rhodiola*’s ability to tolerate high-altitude stress by helping to maintain mitochondrial function under conditions of limited oxygen and other stressors. Although this needs to be confirmed by subsequent experiments, our study shed new light on the subsequent functional exploration of *matR*.

## Conclusion

Comparative genomic analyses showed that the plastomes and mitogenomes of three *Rhodiola* species exhibited contrasting evolutionary pattern, with the former possessing more conserved genome structure but faster evolutionary rates of sequence, whereas the latter exhibiting structural diversity but slower rates of sequence evolution. Lineage-specific features were observed in three *Rhodiola* mitogenomes, including chromosome fission, gene loss and structural rearrangement. We found that in *R. sacra* and *R. wallichiana*, most repetitive sequences occur between the two chromosomes and possess high sequence similarity. This suggests that these repeats may mediate the formation of multichromosomal structure in the mitogenomes of *Rhodiola*, and that such multichromosomal structure may have recently formed. Several intact chloroplast-derived PCGs were identified in their mitogenomes, whereas no intact mitochondrial-derived PCGs were identified in their plastomes, suggesting the presence of intracellular DNA transfer events from chloroplast to mitochondria in *Rhodiola* species. The organelle genomes of three *Rhodiola* species contained multiple fragments of nuclear TEs and exhibit different preferences for TEs insertion type, among which plastomes prefer DNA transposons, while mitogenomes prefer retrotransposons. Signals of positive selection were identified in the gene *matR* of the *Rhodiola* mitogenomes, suggesting that this gene may participate in the adaptive response of *Rhodiola* species to environmental stress of QTP. The genomic data presented herein supplement the genetic knowledge available for the genus *Rhodiola*, and provide valuable insights into the genome evolution of *Rhodiola* species.

## Materials and methods

### Plant materials collection, DNA extraction and sequencing

Three *Rhodiola* species were collected from the QTP at the altitude of 3,540 m (*R. wallichiana*) (sample location: 85.24◦ E, 28.55◦ N), 4,697 m (*R. sacra*) (sample location: 86.37◦ E, 29.49◦ N) and 4,898 m (*R. crenulata*) (sample location: 92.37◦ E, 29.78◦ N). The plant samples were identified by Professor Xing Liu from the Wuhan University according to their morphological characteristics. Total genomic DNA was extracted from their leaves by CTAB method (for Illumina short-read sequencing) and SDS method (for Nanopore ultra-long sequencing). Specially, short-read sequencing was performed using the Illumina HiSeq 4000 platform (Illumina, San Diego CA, USA) with a read length of 150 bp and an insert size of about 350 bp, while the ultra-long sequencing was conducted on a Nanopore PromethION sequencer (Oxford Nanopore, Oxford, UK) with an average read length > 50 kb. All library construction and sequencing steps were completed by Wuhan GrandOmics BioSciences (Wuhan, China).

### Organelle genome assembly and annotation

Because the structure of the plastome is conserved and its assembly technology is relatively mature, only short-read sequencing data was used to assemble the plastome in this study. Three *Rhodiola* plastomes were all de novo assembled using GetOrganelle v1.7.5.3 software [67] with the k-mer length of 21,45,65,85,105 and other default settings. To obtain the complete mitogenomes of three *Rhodiola* species with high accuracy, ultra-long sequencing data was used to assemble the mitogenomes, and Illumina short-read sequencing data was used for base correction. The ultra-long reads were de novo assembled into primary contigs using NextDenovo v2.2 (https://github.com/Nextomics/NextDenovo) with the estimate genome size of 5 mb. Then, we selected contigs with homology to the mitogenomes of *Malus domestica* (NC_018554), *Ziziphus jujuba* (NC_029809) and *Cannabis sativa* (NC_029855) (we chose these three species because they all belong to the order Rosales), and retained contigs with at least one ≥ 5 kb alignment to these mitogenome sequences by BlastN. We then proceeded to align back the ultra-long reads to our mitogenome assembly with minimap2 [68], segregate aligned reads and re-perform de novo assembly using NextDenovo. The final genome sequences of three *Rhodiola* species were obtained by polishing with NextPolish [69] using Illumina short reads. For genome annotation, the plastomes of three *Rhodiola* species were annotated using GeSeq [70], and further checked manually by comparison with the plastomes of *R. kirilowii* (NCBI accession number MT937183), *R. ovatisepala* (MN794328) and *R. rosea* (NC_041671). The mitogenomes of three *Rhodiola* species were annotated using MITOFY [51], and further manually checked by comparison with the mitogenomes of *Malus domestica*, *Ziziphus jujuba* and *Cannabis sativa*. All tRNA genes were predicted using tRNAscan-SE [71]. Ultimately, the organelle genomes of three *Rhodiola* species were visualized using OGDRAW [72].

### Phylogenetic tree reconstruction

Limited by the available mitochondrial genome data of *Rhodiola* species, we were unable to reconstruct the phylogenetic relationships of *Rhodiola* species based on mitochondrial data. Therefore, in this study, we only used chloroplast data to reconstruct the phylogenetic relationships of *Rhodiola* species. The phylogenetic tree of *Rhodiola* species was reconstructed using protein-coding genes of plastomes from 20 *Rhodiola* species and two outgroups (*Phedimus aizoon* and *Hylotelephium erythrostictum*). The genera in which these two outgroups belong are closely related to *Rhodiola* [24]. After removing one of the two copies of the genes in the inverted repeat region, the 79 shared protein-coding genes of 22 plastomes were extracted, aligned separately, and recombined to construct a super-matrix using PhyloSuite_v1.2.2 [73]. Subsequently, the super-matrix was used to conduct the Bayesian inference (BI) and Maximum likelihood (ML) phylogenies. The BI tree was inferred using MrBayes 3.2.6 [74] under JC + I + G model, which was determined from the ModelFinder [75]. The ML tree was inferred using IQ-TREE [76] under an edge-linked partition model for 5,000 ultrafast bootstraps, as well as the Shimodaira–Hasegawa-like approximate likelihood-ratio test.

### Collinearity analysis, identification of homologous sequences and transposable elements

To identify possible structural rearrangements, the nucmer program of MUMmer [77] was used to align the plastomes of three *Rhodiola* species. Then, Mummerplot was used to visualize the alignment results produced by mummer using the GNU gnuplot utility. Collinearity regions among mitogenomes of three *Rhodiola* species were identified using BLASTn plugin from TBtools [78] with the following parameters: matching rate ≥ 80% and E-value ≤ 1e-5. Then, the R package RIdeogram [79] was used to visualize the collinearity regions with default settings. Repetitive sequences within the genome were identified using BLASTn with an E-value ≤ 1e-5, and the results were visualized using the “advanced circos” function in TBtools [78]. Likewise, to detect the possible sequences transformation between plastome and mitogenome, homologous sequences were also identified using BLASTn. Nuclear TEs in the organelle genomes of three *Rhodiola* species were identified using CENSOR web server [80] with default parameters and ‘green plants’ as a reference sequence source.

### Estimation of nucleotide substitution rate

To facilitate comparisons of nucleotide substitution rate in three *Rhodiola* species, we have roughly obtained the mitogenome sequence of an outgroup *Hylotelephium erythrostictum* by mapping its Illumina raw reads (download from GenBank database under the accession number SRR15239599) to the mitogenome sequence of *R. crenulata*. We then annotated and extracted 20 complete PCGs from the draft mitogenome of *Hylotelephium erythrostictum*, which were regarded as references for calculating nucleotide substitution rates of PCGs in the three *Rhodiola* mitogenomes. Totally, the organelle genomes of three *Rhodiola* species (*R. wallichiana*, *R. crenulata* and *R. sacra*) were used, and *Hylotelephium erythrostictum* was included as an outgroup. We processed 79 shared PCGs from plastomes and 18 shared PCGs from mitogenomes in parallel using PhyloSuite software. Synonymous (dS) and nonsynonymous (dN) substitution rates were calculated using KaKs_Calculator [81] with the MLWL model. To examine significant differences between gene sets, analyses of variance with Wilcoxon paired test were performed using R.

Then, the branch-site model was applied to estimate the selective pressure presumably caused by environmental adaptation using the PAML software [82]. The tree topology of the three *Rhodiola* species and an outgroup *Hylotelephium erythrostictum* was derived from the phylogenetic results in Sect. 2.3. We used the codeml program’s branch-site model to identify positively selected genes in the foreground branch (three *Rhodiola* species). The P-value of LRT was acquired by the Chi-squared test, and the BEB method was implemented to test sites that are potentially under positive selection. Subsequently, genes with p < 0.05 and ω > 1 were selected as candidate positively selected genes.

## Electronic supplementary material

Below is the link to the electronic supplementary material.

Supplementary Material 1

Supplementary Material 2

Supplementary Material 3

Supplementary Material 4

Supplementary Material 5

Supplementary Material 6

Supplementary Material 7

Supplementary Material 8

Supplementary Material 9

Supplementary Material 10

Supplementary Material 11

Supplementary Material 12

Supplementary Material 13

Supplementary Material 14

## Data Availability

The data that support the findings of this study are openly available in the GenBank database under accession numbers OP312064 (*R. crenulata*), OP312065 (*R. wallichiana*) and OP312066 (*R. sacra*), and the mitogenome sequences generated in this study are deposited in GenBank database under accession numbers OP312067 (*R. crenulata*), OP312068 (*R. wallichiana* chromosome 1), OP312069 (*R. wallichiana* chromosome 2), OP312070 (*R. sacra* chromosome 1), and OP312071 (*R. sacra* chromosome 2). The organelle genomes of three *Rhodiola* species are available in figshare database (10.6084/m9.figshare.20657442.v1) [83].
