# Supplementary material for: Comparative analysis of the organelle genomes of three Rhodiola species provide insights into their structural dynamics and sequence divergences
Source: BMC Plant Biol. 2023 Mar 22;23:156. doi: 10.1186/s12870-023-04159-1 (PMC10031898; doi:10.1186/s12870-023-04159-1)
Supplement: Supplementary file 1 — Supplementary Material 1 [file 12870_2023_4159_MOESM1_ESM.docx]

**Figure S1.** The mapping coverage of three *Rhodiola* plastomes based on Illumina reads. Green represents the gene, yellow represents the coding region of the gene, and red represents the tRNA. (A) *R. crenulata*. (B) *R. sacra* chromosome 1. (C) *R. sacra* chromosome 2. (D) *R. wallichiana* chromosome 1. (E) *R. wallichiana* chromosome 2.

**Figure S2.** Syntenic regions between *R. crenulata* and *R. sacra* plastomes.
