# Supplementary figures and images for: Comparative analysis of the organelle genomes of three Rhodiola species provide insights into their structural dynamics and sequence divergences
Source: BMC Plant Biol. 2023 Mar 22;23:156. doi: 10.1186/s12870-023-04159-1 (PMC10031898; doi:10.1186/s12870-023-04159-1)

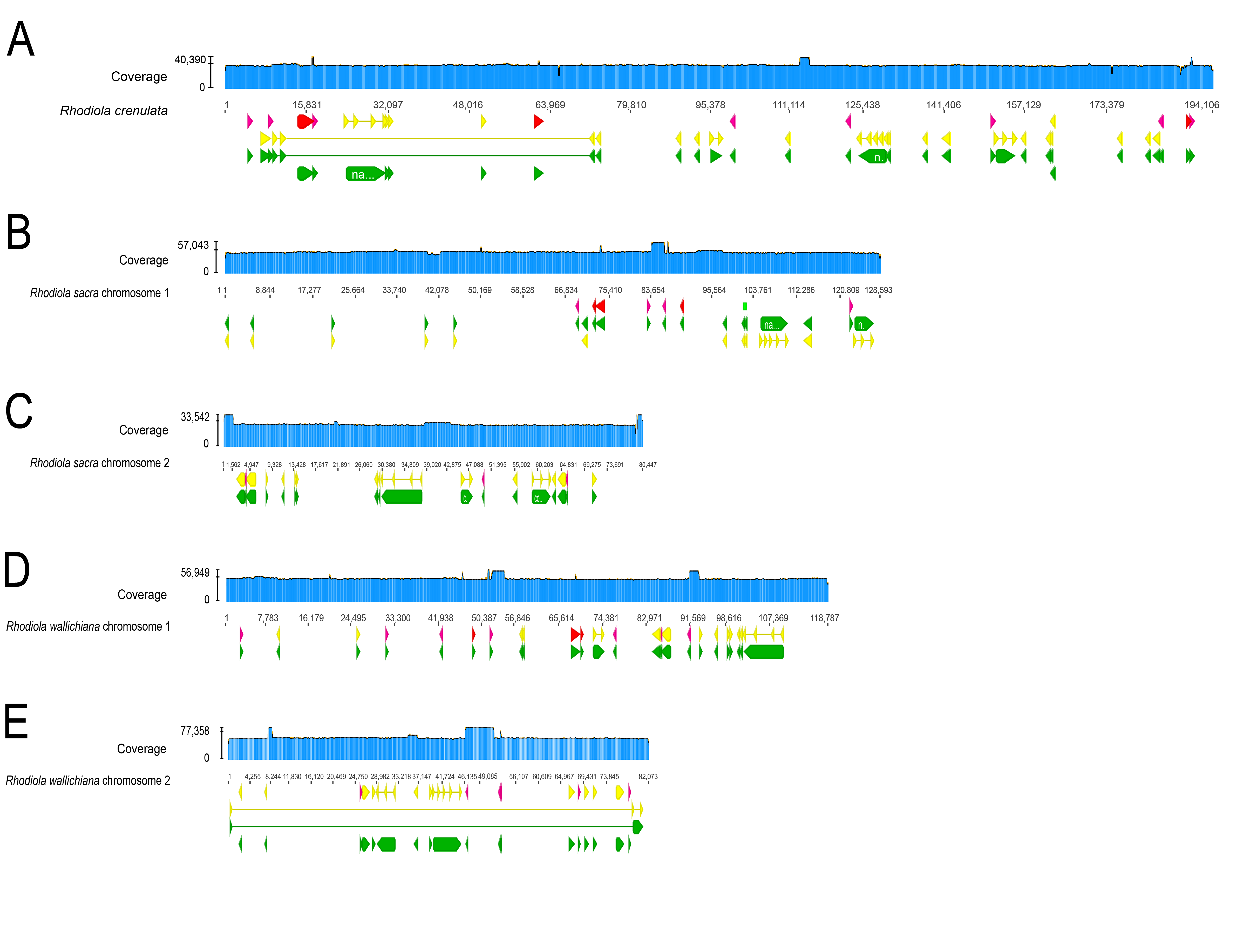

Supplement: Supplementary file 2 — Supplementary Material 2 [file 12870_2023_4159_MOESM2_ESM.png]

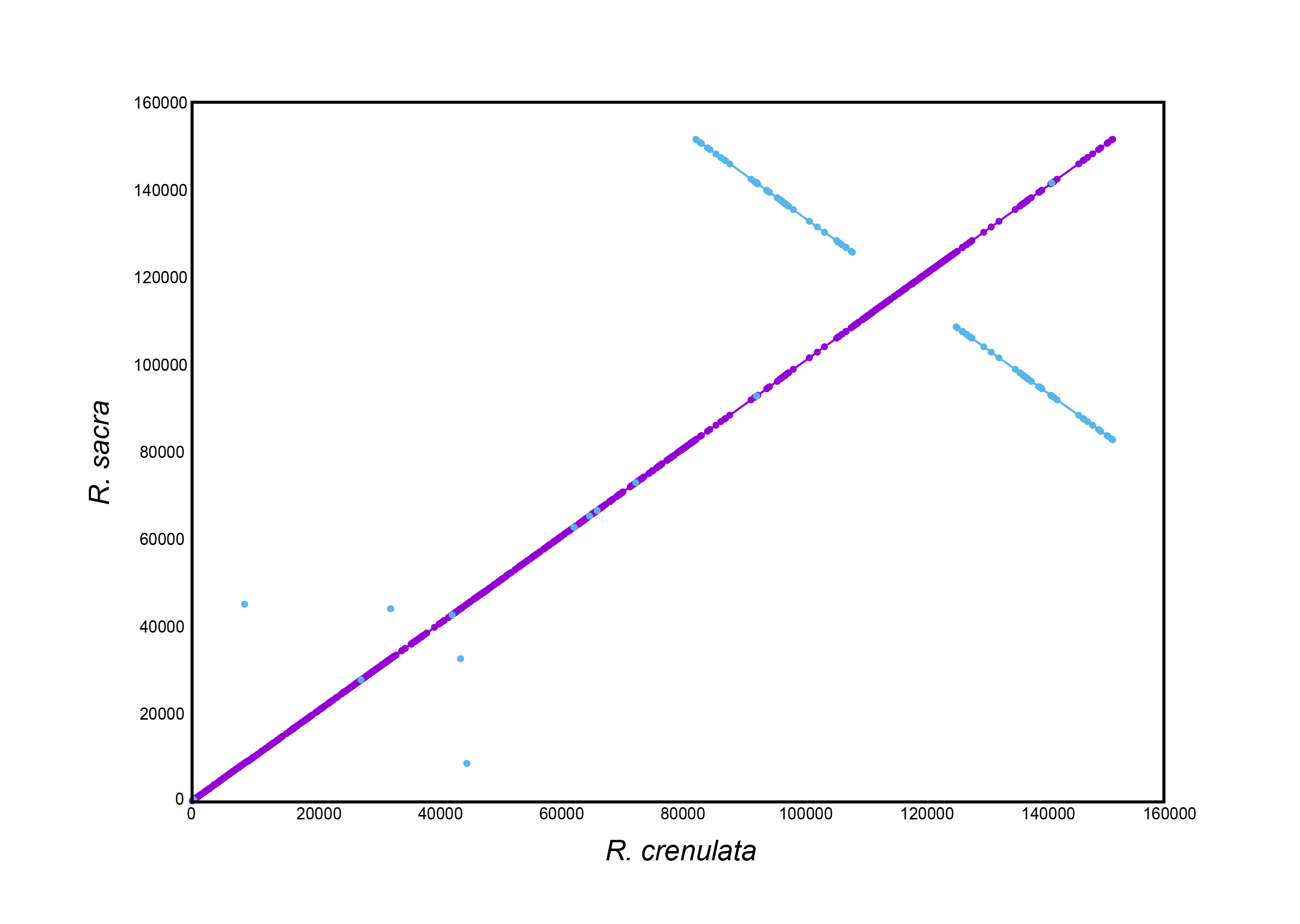

Supplement: Supplementary file 3 — Supplementary Material 3 [file 12870_2023_4159_MOESM3_ESM.png]
