# Supplementary material for: Comparative analysis of the organelle genomes of three Rhodiola species provide insights into their structural dynamics and sequence divergences
Source: BMC Plant Biol. 2023 Mar 22;23:156. doi: 10.1186/s12870-023-04159-1 (PMC10031898; doi:10.1186/s12870-023-04159-1)
Supplement: Supplementary file 4 — Supplementary Material 4 [file 12870_2023_4159_MOESM4_ESM.docx]

Table S1. Gene annotation of the *Rhodiola* chloroplast genomes.

| **Category** | **Group** | **Genes** |
| --- | --- | --- |
| Photosynthesis related genes | Rubisco | *rbcL* |
|  | Photosystem I | *psaA, psaB, psaC, psaI, psaJ* |
|  | Photosystem II | *psbA, psbB, psbT, psbK, psbI, psbH, psbM, psbN, psbD, psbC, psbZ, psbJ, psbL, psbE, psbF* |
|  | ATP synthase | *atpA, atpB, atpE, atpF^a^, atpH, atpI* |
|  | Cytochrome b/f complex | *petA, petB^b^, petD^a^, petN, petL, petG* |
|  | Cytochrome csynthesis | *ccsA* |
|  | Complex I of chloroplasts | *ndhA^a^, ndhB^a,c^, ndhC, ndhD, ndhE, ndhF, ndhH, ndhG, ndhJ, ndhK, ndhI* |
| Transcription and translation related genes | Transcription | *rpoA, rpoB, rpoC2, rpoC1^a^,* |
|  | Ribosomal proteins | *rps2, rps3, rps4, rps7^c^, rps8, rps11, rps12^b,c^, rps14, rps15, rps16^a^, rps18, rps19, rpl2*^a,c^*, rpl14, rpl16^a^, rpl22, rpl23^c^, rpl32, rpl33, rpl36* |
|  | Translation initiation factor | *infA* |
| RNA genes | Ribosomal RNA | *rrn16S^c^, rrn23S^c^, rrn4.5^c^, rrn5^c^* |
|  | Transfer RNA | *trnH-GUG, trnK-UUU^a^, trnQ-UUG, trnS-GCU, trnS-UGA, trnS-GGA*, *trnR-UCU, trnR-ACG^c^*, *trnC-GCA, trnD-GUC, trnY-GUA, trnE-UUC, trnT-UGU*, *trnfM-CAU, trnL-CAA^c^, trnL-UAA^a^, trnL-UAG, trnF-GAA, trnV-GAC^c^, trnV-UAC^a^, trnM-CAU, trnW-CCA, trnP-UGG, trnI-CAU^c^, trnI-GAU*^a,c^*, trnA-UGC*^a,c^*, trnN-GUU^c^* |
| Other genes | RNA processing | *matK* |
|  | Carbon metabolism | *cemA* |
|  | Fatty acid synthesis | *accD* |
|  | Proteolysis | *clpP^b^* |
|  | Conserved ORFs | *ycf1, ycf2^c^, ycf3^b^, ycf4* |

^a^genes with one intron, ^b^genes with two introns, ^c^Two gene copies in IRs.
