# Supplementary material for: Comparative analysis of the organelle genomes of three Rhodiola species provide insights into their structural dynamics and sequence divergences
Source: BMC Plant Biol. 2023 Mar 22;23:156. doi: 10.1186/s12870-023-04159-1 (PMC10031898; doi:10.1186/s12870-023-04159-1)
Supplement: Supplementary file 5 — Supplementary Material 5 [file 12870_2023_4159_MOESM5_ESM.docx]

Table S2. Gene content of the *R. crenulata* mitogenome.

| **Category** | **Group** | **Genes** |
| --- | --- | --- |
| Mitochondrial respiratory chain related genes | Complex I | *nad2^b^, nad4^b^, nad4L, nad5^b^, nad6, nad7^b^, nad9* |
|  | Complex II | *sdh4* |
|  | Complex IV | *cox1, cox3* |
|  | Complex V | *atp1, atp4, atp6, atp8* |
|  | Cytochrome c synthesis | *ccmB, ccmC, ccmFc^a^* |
| Transcription and translation related genes | Ribosomal proteins | *rpl10, rpl5, rps13* |
| RNA genes | Transfer RNA | *trnM-CAU, trnE-UUC, trnH-GUG, trnW-CCA, trnY-GUA* |
|  | Ribosomal RNA | *rrn5, rrn18, rrn26* |
| Other genes | Maturase | *matR* |
|  | Methyltransferase | *mttB* |

^a^genes with one intron, ^b^genes with at least two introns.
