# Supplementary material for: Comparative analysis of the organelle genomes of three Rhodiola species provide insights into their structural dynamics and sequence divergences
Source: BMC Plant Biol. 2023 Mar 22;23:156. doi: 10.1186/s12870-023-04159-1 (PMC10031898; doi:10.1186/s12870-023-04159-1)
Supplement: Supplementary file 6 — Supplementary Material 6 [file 12870_2023_4159_MOESM6_ESM.docx]

Table S3. Gene content of the *R. sacra* mitogenome.

| **Category** | **Group** | **Chr1** | **Chr2** |
| --- | --- | --- | --- |
| Mitochondrial respiratory chain related genes | Complex I | *nad2^b^, nad7^b^, nad9* | *nad3, nad4^b^, nad4L* |
|  | Complex III | *cob* | */* |
|  | Complex IV | *cox1, cox3* | *cox2^a^,* |
|  | Complex V | *atp8* | *atp1, atp4, atp6,* |
|  | Cytochrome c synthesis | *ccmB* | *ccmFn, ccmC, ccmFc^a^* |
| Transcription and translation related genes | Ribosomal proteins | *rpl5, rps13, rps14* | *rpl10, rps4, rps7, rps12* |
| RNA genes | Transfer RNA | *trnM-CAU, trnH-GUG, trnQ-UUG, trnY-GUA* | *trnM-CAU (*×2*), trnE-UUC* |
|  | Ribosomal RNA | *rrn5, rrn18, rrn26* | */* |
| Other genes | Maturase | */* | *matR* |
|  | Methyltransferase | *mttB* | */* |

^a^genes with one intron, ^b^genes with at least two introns.
