# Supplementary material for: Comparative analysis of the organelle genomes of three Rhodiola species provide insights into their structural dynamics and sequence divergences
Source: BMC Plant Biol. 2023 Mar 22;23:156. doi: 10.1186/s12870-023-04159-1 (PMC10031898; doi:10.1186/s12870-023-04159-1)
Supplement: Supplementary file 7 — Supplementary Material 7 [file 12870_2023_4159_MOESM7_ESM.docx]

Table S4. Gene content of the *R. wallichiana* mitogenome.

| **Category** | **Group** | **Chr1** | **Chr2** |
| --- | --- | --- | --- |
| Mitochondrial respiratory chain related genes | Complex I | *nad3, nad4^b^, nad4L* | *nad2^b^, nad7^b^, nad9* |
|  | Complex III | */* | *cob* |
|  | Complex IV | */* | *cox1, cox2^a^, cox3* |
|  | Complex V | *atp4, atp8* | *atp1, atp6* |
|  | Cytochrome c synthesis | *ccmFn, ccmFc^a^* | *ccmB, ccmC* |
| Transcription and translation related genes | Ribosomal proteins | *rpl10, rpl5, rps7, rps12, rps14* | *rps4, rps13* |
| RNA genes | Transfer RNA | *trnM-CAU (*×5*), trnH-GUG, trnY-GUA,* | *trnE-UUC, trnP-UGG, trnQ-UUG, trnW-CCA, trnV-GAC, trnY-GUA* |
|  | Ribosomal RNA | *rrn5, rrn18, rrn26* | */* |
| Other genes | Maturase | *matR* | */* |
|  | Methyltransferase | *mttB* | */* |

^a^genes with one intron, ^b^genes with at least two introns.
